# Sex differences in 10-year cardiovascular risk of patients with type 2 diabetes mellitus and subclinical hypothyroidism: a cross-sectional study

**DOI:** 10.3389/fendo.2025.1635444

**Published:** 2025-07-31

**Authors:** Xiang Zhao, Ke He, Ji Li, Lingyan Zhou, Ling Liu, Xiwan Lu, Yan Jiang

**Affiliations:** ^1^ Department of Graduate School, Nanjing University of Chinese Medicine, Nanjing, China; ^2^ Department of Endocrinology, Wuxi Affiliated Hospital of Nanjing University of Chinese Medicine, Wuxi, China

**Keywords:** subclinical hypothyroidism, type 2 diabetes mellitus, cardiovascular disease risk, sex differences, comorbidity

## Abstract

**Objective:**

To evaluate gender-specific variations in cardiovascular disease (CVD) risk stratification and its modifiable determinants among individuals concurrently diagnosed with type 2 diabetes mellitus (T2DM) and subclinical hypothyroidism (SCH).

**Methods:**

A cross-sectional observational study was conducted involving 2,357 patients with T2DM (1,120 males and 1,237 females) who were hospitalized at Wuxi Hospital of Traditional Chinese Medicine between 2018 and 2024. Participants were categorized into the SCH (n=196) and the euthyroid subgroups (n=2,161). The 10-year probability of cardiovascular events was estimated based on the Framingham Risk Score (FRS) model. Sex-specific differences in SCH prevalence and CVD risk were examined, and associations between FRS and biomarkers—namely thyroid-stimulating hormone (TSH), free thyroxine (FT4), cystatin C (CysC) and other factors—were analyzed via Spearman’s correlation analysis and multivariable linear regression.

**Results:**

The prevalence of SCH in T2DM patients was 9.06% (10.02% in females vs. 6.43% in males). Male patients diagnosed with SCH exhibited an elevated FRS compared to their euthyroid counterparts (21.00 vs. 20.00, *P=* 0.025). Within this subgroup, a positive relationship was identified between TSH levels and FRS(r=0.374, *P=* 0.001), whereas FT4 showed a negative association (*r=*-0.342, *P=* 0.003). These relationships were not statistically significant among women diagnosed with SCH. Cystatin C was positively associated with FRS in both male (*r=*0.461, *P<*0.001) and female (*r=*0.452, *P<*0.001) groups. Multivariable linear regression evaluation in male patients revealed that TSH (*β=*3.87, *P=* 0.048), cystatin C (*β=*1.48, *P=* 0.03), and FT4 (*β=*-0.61, *P=* 0.011) continued to be significantly correlated with 10-year CVD risk. Additionally, male patients with SCH exhibited significantly higher smoking status, uric acid, and creatinine levels than their female counterparts (all *P<*0.05), indicating that sex-specific risk factors may contribute to elevated CVD risk.

**Conclusion:**

This study identified higher FRS in male versus female patients with comorbid T2DM and SCH, potentially mediated by sex-specific variations in TSH, FT4, and CysC levels. These results underscore the importance of implementing sex-specific strategies for CVD risk management in this population.

## Graphical Abstract

Graphical abstract of the study. Graphical abstract illustrating the sex-specific cardiovascular risk associated with type 2 diabetes mellitus (T2DM) and subclinical hypothyroidism (SCH). Biochemical indicators—including TSH, FT4, CysC, and others—were analyzed using Python 3.10. The results highlight the need for routine monitoring of TSH and CysC in male patients with T2DM and SCH, and for prioritizing cardiovascular risk stratification in this population. The asterisk (*) highlights that the FRS is higher in male patients with comorbid diabetes mellitus and subclinical hypothyroidism compared to the other three groups.

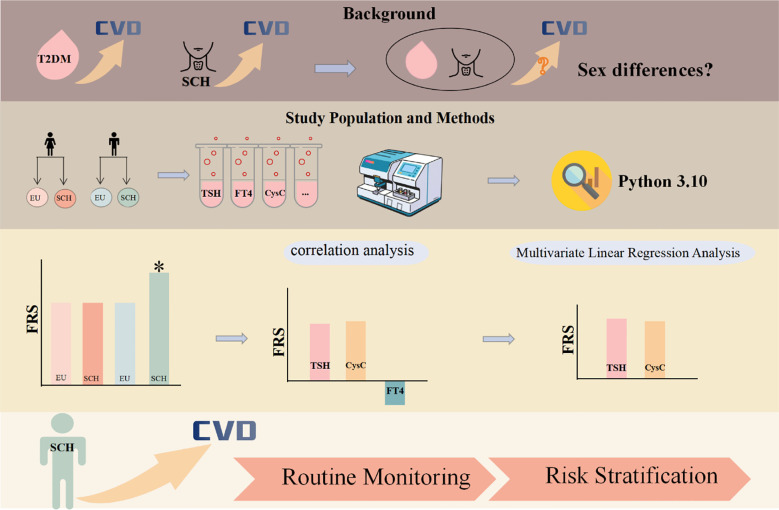

## 1 Introduction

Subclinical hypothyroidism (SCH) is characterized by a mild elevation in serum thyroid-stimulating hormone (TSH) levels, accompanied by free thyroxine (FT4) and triiodothyronine (FT3)concentrations that remain within their respective reference ranges. SCH has been implicated as a possible contributor to increased cardiovascular disease (CVD) risk (1).

As a systemic disorder of glucose metabolism, diabetes mellitus (DM) manifests through chronic hyperglycemia caused by various endocrine and metabolic disturbances. Cardiovascular disease continues to be the primary cause of mortality in patients with T2DM, contributing to nearly 50.3% of total deaths in this group (2). Furthermore, individuals with DM exhibit an elevated prevalence of SCH compared with the general population (3). The comorbidity of DM and SCH may further elevate the risk of CVD, and the underdiagnosis or underrecognition of either condition may exacerbate this risk. Therefore, particular attention should be directed toward cardiovascular risk among individuals presenting with both DM and SCH. While existing research has assessed the relationship between DM-SCH comorbidity and cardiovascular outcomes, the results remain inconclusive. Some investigations have reported a markedly increased incidence of coronary heart disease among patients with type 2 diabetes and SCH, relative to euthyroid counterparts (4). In contrast, previous research did not establish a clear association between SCH and the occurrence of CVD events or mortality in this population (5). Given the uncertainties, additional research is required to better clarify how the comorbidity of DM and SCH influences cardiovascular risk and disease progression.

Framingham Risk Score (FRS) is an authority for assessing the risk of cardiovascular disease (6),which is effective in predicting cardiovascular risk and guiding the development of preventive measures (7, 8). However, few studies have specifically examined FRS characteristics and gender-related differences in individuals with coexisting DM and SCH. Given the ongoing debate regarding the cardiovascular risk associated with DM and SCH comorbidity, the present study seeks to address this gap through a cross-sectional analysis. Specifically, the aims of this work are to: (1) evaluate the independent association between SCH and FRS; (2) examine gender differences in FRS among patients with DM and SCH; (3) explore the interaction between thyroid function markers (e.g., TSH) and other clinical parameters with FRS. Findings from this study may help inform precise thyroid function management in patients with comorbid DM and SCH.

## 2 Materials and methods

### 2.1 Data source

The acquisition of clinical data for this work was conducted through the “Weiyun Medical Intelligent Research Data Service Platform,” which includes comprehensive clinical information of patients hospitalized at Wuxi Hospital of Traditional Chinese Medicine between 2015 and 2024. This database comprises demographic characteristics, disease diagnoses, prescription records, laboratory test results, radiological reports, and clinical notes. All patient information in the database was anonymized. The study adhered to the ethical principles set forth in the Declaration of Helsinki (9) and received approval from the Ethics Committee of Wuxi Hospital of Traditional Chinese Medicine (Approval No. 2025(Research)-053-01).

### 2.2 Diagnostic criteria

Diabetes mellitus was diagnosed in accordance with the 2020 edition of the Chinese Guidelines for the Prevention and Treatment of Type 2 Diabetes. Diabetes mellitus was diagnosed if the patient exhibited typical symptoms (e.g., excessive thirst, polydipsia, polyphagia, unexplained weight loss) and met any of the criteria listed below:

1. random plasma glucose level equal to or exceeding 11.1 mmol/L;
2. fasting plasma glucose concentration of 7.0 mmol/L or higher (fasting was characterized by the absence of caloric consumption for at least 8 hours);
3. A post-load plasma glucose concentration equal to or exceeding 11.1 mmol/L measured two hours after an oral glucose tolerance test (OGTT);
4. A glycated hemoglobin (HbA1c) level of no less than 6.5%.

For patients without typical symptoms, diagnosis required confirmation of abnormal glucose or HbA1c on a separate day.

Subclinical hypothyroidism is biochemically defined by increased serum thyroid-stimulating hormone (TSH) levels alongside FT3 and FT4 concentrations that remain within the physiological reference range (10).

### 2.3 Inclusion criteria

1. Age ≥30 years (according to the Framingham Risk Score criteria);
2. Fulfilled diagnostic criteria for diabetes mellitus;
3. Demonstrated either subclinical hypothyroidism or normal thyroid function;
4. Availability of complete clinical data;

### 2.4 Exclusion criteria

1. Severe hepatic or renal dysfunction, malignancies, hematologic or immune disorders, acute or chronic infections, recent trauma or surgery, or acute/severe chronic diabetic complications
2. A prior diagnosis of thyroid disorders or administration of agents known to influence thyroid function (e.g., levothyroxine, methimazole, propylthiouracil, amiodarone, NSAIDs, hormonal therapies);
3. Documented cardiovascular or cerebrovascular diseases, including coronary heart disease, transient ischemic attack, cerebral infarction, cerebral hemorrhage, or subarachnoid hemorrhage;
4. Pregnancy or uremia.

### 2.5 Data acquisition

#### 2.5.1 Demographic and clinical characteristics

Clinical variables were extracted from patient medical records, including the following information: (1)general data including age, sex, family history of coronary heart disease, smoking and alcohol use, anthropometric measurements (e.g., height, weight, and BMI), and blood pressure readings (systolic and diastolic values); (2)current medication types, including: ①antihyperglycemic agents: GLP-1 receptor agonists, metformin, DPP-4 inhibitors, gliclazide, α-glucosidase inhibitors, PPARγ agonists, Inhibitors of the sodium-glucose cotransporter-2(SGLT-2), sulfonylureas, and insulin; ②antihypertensive agents: agents including angiotensin-converting enzyme inhibitors (ACEIs), angiotensin II receptor blockers (ARBs), β-adrenergic blockers, and calcium channel antagonists, α-receptor antagonists, and diuretics; ③antiplatelet drugs: aspirin, clopidogrel; ④ lipid-lowering agents: statins, ezetimibe, and fibrates; (3)comorbidities: hypertension and hepatic steatosis.

#### 2.5.2 Biochemical assessment

Morning venous samples were obtained following an overnight fast. The Beckman Coulter AU5800 system was employed to measure various metabolic markers: fasting plasma glucose (FPG), uric acid (UA), renal function parameters (creatinine [Cr] and cystatin C [CysC]), lipid profile components (cholesterol [CHO], triglycerides [TG], LDL-C, HDL-C, and lipoprotein(a) [Lp(a)]), along with high-sensitivity C-reactive protein (hs-CRP). The atherosclerosis index (AI) was determined by the equation: (CHO minus HDL-C) divided by HDL-C. Glycated hemoglobin (HbA1c) was measured using the Bio-Rad VARIANT™ II system. Thyroid function was assessed via chemiluminescent immunoassay (Cobas e602, Roche), and the measured hormone levels were interpreted based on the following reference intervals provided by the testing laboratory: TSH (0.27–4.2 μIU/mL), FT4 (12–22 pmol/L), and FT3 (3.1–6.8 pmol/L).

#### 2.5.3 Evaluation of cardiovascular risk

Cardiovascular risk over the subsequent decade was quantified using the Framingham Risk Score (FRS), which estimates the probability of a first event based on established clinical parameters. This model applies sex-specific Cox proportional hazards regression equations incorporating multiple cardiovascular risk factors, including age, levels of total and HDL-C, SBP, antihypertensive medication use, smoking behavior, and presence or absence of diabetes. The absolute cardiovascular risk was derived from these parameters as described previously (11).

### 2.6 Database creation and data preprocessing

The database compiles diabetes patients’ diagnosis and treatment data of Wuxi Hospital of Traditional Chinese Medicine from 2015 to 2024, and the data scope includes including patient demographics, physical examination results, diagnostic records, laboratory and imaging reports, inpatient and outpatient prescriptions, surgical records, and clinical course documentation. Big data governance technologies are employed to clean, transform, desensitize, and standardize the medical records. These processed data are then integrated into a specialized database for diabetes, enabling systematic storage and analysis of disease-specific information.

### 2.7 Statistical analysis

Participants were stratified by sex into male and female groups, each further subdivided into SCH and euthyroid subgroups for comparative analyses between subgroups. The 10-year cardiovascular risk was calculated accordingly. The normal distribution of the data was assessed utilizing the Kolmogorov-Smirnov test along with the Shapiro-Wilk test. Depending on data type and distribution, appropriate statistical methods, including independent sample t-tests, chi-square analyses, and non-parametric approaches, were employed to evaluate differences in baseline demographic and clinical variables, and FRS between SCH and euthyroid subgroups stratified by sex. Quantitative variables following a normal distribution are presented using the mean and standard deviation (mean ± SD), while qualitative variables are conveyed as counts (n) alongside their corresponding percentages (%). Spearman correlation analysis was utilized to explore relationships between FRS and thyroid-related hormones (TSH, FT3, FT4), HbA1c, UA, Cr, CysC, LDL-C, HDL-C, atherosclerosis index, and hs-CRP. Variables significantly associated with FRS were subsequently entered into linear regression analyses. To verify the robustness of the results, adjustments were made for potential confounders, including alcohol consumption, use of statins, ezetimibe, SGLT2 inhibitors, insulin, and HbA1c. Given that CysC serves as a highly sensitive indicator of renal status, and renal dysfunction has the potential to alter cardiovascular risk profiling outcomes, potential confounding effects due to comorbid chronic kidney disease (CKD) were considered. A review of the dataset revealed that none of the patients with SCH had coexisting CKD, as identified by a calculated glomerular filtration rate (eGFR) below 60 mL/min/1.73 m²(n=0), thereby excluding the possibility of confounding from CKD. Python version 3.10 was utilized to carry out all statistical computations.

### 2.8 Research design process

**Figure 1:**
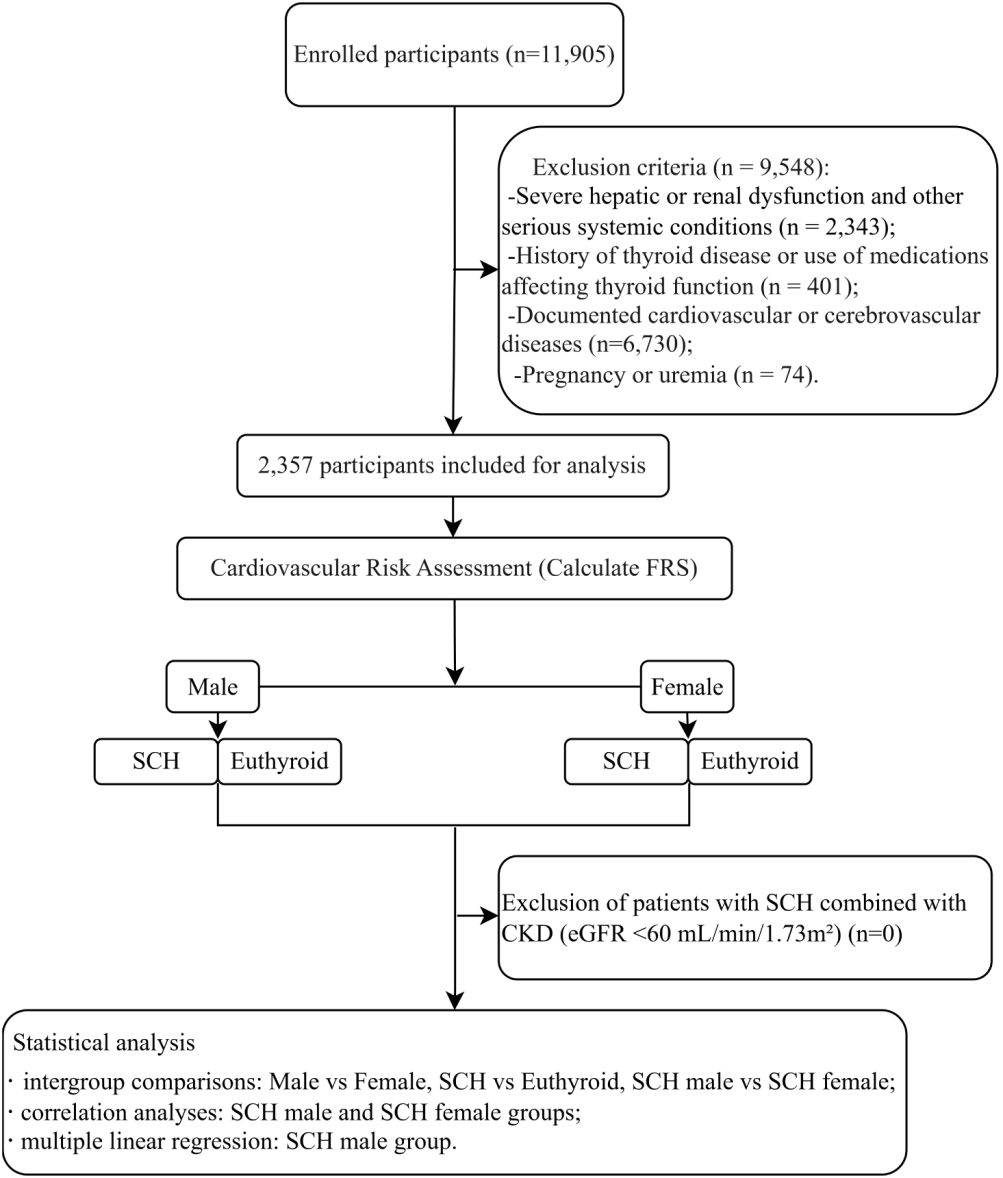
Diagram illustrating the research protocol. Hospitalized patients with diabetes at Wuxi Hospital of Traditional Chinese Medicine between 2018 and 2024 were screened, and a total of 1,120 male and 1,237 female patients meeting the inclusion and exclusion criteria were enrolled. These patients were further categorized into a SCH)group and a euthyroid group for subsequent analyses.

The flowchart of this research design is presented in **Figure 1**.

## 3 Results

### 3.1 Analysis of demographic and clinical variables by SCH status stratified by sex

The study ultimately included 2,357 patients diagnosed with DM, comprising 1,120 men and 1,237 women. SCH occurred in 9.06% (72/1,120) of the diabetic population included in the study. Notably, the prevalence was higher in female patients (10.02%, 124/1,237) compared to male patients (6.43%, 67/1,147).

In the male group, the levels of serum TSH and cystatin C (both *P<*0.001), LDL-C (*P=* 0.001),and creatinine (*P=* 0.031) were notably higher in patients with SCH compared to those without. Conversely, among patients with SCH, HbA1c, FT4, and HDL-C levels were markedly decreased, with P-values of 0.01, 0.001, and <0.001, respectively. Furthermore, a greater percentage of individuals in the SCH group were prescribed diuretics (*P=* 0.004), whereas insulin (*P=* 0.031) and SGLT-2 inhibitor (*P=* 0.039) prescriptions were notably less common compared to the non-SCH group. In the female group, serum TSH levels were markedly higher in patients with SCH (*P<*0.001), while serum FT4 (*P<*0.001) and LDL-C (*P=* 0.048) levels were significantly lower than in their non-SCH counterparts. In addition, the use of α-glucosidase inhibitors was more common among female patients with SCH (*P=* 0.001) (**Table 1**).

**Table 1:** Comparison of baseline demographic and clinical data between diabetic patients with SCH and those with euthyroid controls.

Variables	Male		P value	Female		P value
	With SCH (N=72)	Without SCH (N=1048)		With SCH (N=124)	Without SCH (N=1113)	
FT4 (pmol/L)	15.8475 ± 2.2585	16.8232 ± 2.4909	0.001**	15.47 (14.25,16.71)	16.23 (14.78,17.79)	<0.001***
TSH (μIU/mL)	5.89 (5.13,6.91)	1.54 (1.07,2.24)	<0.001***	5.40 (4.72,6.51)	1.86 (1.24,2.60)	<0.001***
CysC (mg/L)	1.05 (0.83,1.41)	0.87 (0.77,1.01)	<0.001***	0.82 (0.71,1.02)	0.85 (0.74,0.99)	0.621
Cr (μmol/L)	72.25 (60.90,100.00)	67.80 (54.77,89.15)	0.031*	53.65 (44.83,68.25)	52.50 (46.20,66.00)	0.767
LDL-C (mmol/L)	3.1886 ± 1.0677	2.8356 ± 0.8759	0.001**	2.92 (2.17,3.34)	2.99 (2.41,3.52)	0.048*
HDL-C (mmol/L)	0.85 (0.75,1.02)	1.07 (0.91,1.22)	<0.001***	1.16 (1.02,1.43)	1.19 (1.04,1.38)	0.79
AGI	25 (34.72%)	347 (33.11%)	0.779	60 (48.39%)	365 (32.79%)	0.001**
SGLT_2	5 (6.94%)	168 (16.03%)	0.039*	10 (8.06%)	139 (12.49%)	0.151
Insulin	10 (13.89%)	264 (25.19%)	0.031*	22 (17.74%)	189 (16.98%)	0.831
Diuretic	10 (13.89%)	58 (5.53%)	0.004**	8 (6.45%)	82 (7.37%)	0.71
HbA1c (%)	7.85 (6.78,8.49)	8.49 (7.10,9.62)	0.01*	7.95 (6.67,8.49)	8.49 (7.00,8.70)	0.059
FRS	21.00 (16.75,24.00)	20.00 (17.00,22.00)	0.025*	20.00 (16.00,22.00)	20.00 (17.00,22.00)	0.261

FT4, free thyroxine; TSH, Thyroid-Stimulating Hormone; CysC, Cystatin C; Cr, Creatinine; LDL-C/HDL-C, (low/high-density lipoprotein-cholesterol); AGI, Alpha-glucosidase inhibitors; SGLT_2, Sodium-glucose cotransporter protein 2 inhibitor; HbA1c, Glycosylated Hemoglobin, Type A1C. *P<0.05, **P<0.01, ***P<0.001. Values with *P* < 0.05 were interpreted as statistically significant.

Significant differences between male and female patients with SCH were observed in serum TSH (*P=* 0.026), CysC, height, weight, and Cr (all *P<*0.001), as well as smoking status and serum FT3 (both *P=* 0.001), and UA (*P=* 0.007). Additionally, LDL-C and FRS were markedly higher in female patients (both *P=* 0.018). HDL-C (*P=* 0.001) and CHO (*P=* 0.008) were markedly lower among male individuals with SCH compared to their female counterparts (**Table 2**).

**Table 2:** Analysis of baseline demographic and clinical features between male and female diabetic patients, and between male and female patients with SCH.

Variables	Male (N=1120)	Female (N=1237)	P value	Male with SCH (N=72)	Female with SCH (N=124)	P value
Age (years)	58.00 (50.00,68.00)	64.00 (56.00,72.00)	<0.001***	61.8194 ± 14.0245	61.8871 ± 11.6795	0.971
Alcohol consumption	195 (17.41%)	7 (0.57%)	0.001**	–	–	–
Smoking	383 (34.20%)	22 (1.78%)	0.001**	18 (25.00%)	1 (0.81%)	0.001**
height (cm)	170.00 (168.00,175.00)	160.00 (156.00,163.00)	<0.001***	170.0972 ± 5.7339	159.3629 ± 5.1345	<0.001***
weight (kg)	71.00 (65.00,78.00)	61.54 (55.00,68.81)	<0.001***	70.61 (63.75,76.50)	62.00 (55.00,67.00)	<0.001***
BMI (kg/m^2^)	24.45 (22.49,26.37)	24.09 (22.22,26.35)	0.044*	25.0047 ± 4.5394	24.0812 ± 3.4409	0.11
SBP (mmHg)	133.00 (124.75,143.25)	138.00 (126.00,150.00)	<0.001***	138.7488 ± 23.4463	139.4124 ± 20.6847	0.837
DBP (mmHg)	80.00 (76.00,90.00)	80.00 (75.00,86.00)	0.009**	82.74 (75.00,90.00)	80.00 (76.75,90.00)	0.627
Hypertension	560 (50.00%)	698 (56.43%)	0.002**	34 (47.22%)	63 (50.81%)	0.629
FT3 (pmol/L)	4.53 (4.05,4.96)	4.20 (3.76,4.66)	<0.001***	4.5510 ± 0.5837	4.2594 ± 0.6006	0.001**
FT4 (pmol/L)	16.59 (15.11,18.32)	16.19 (14.70,17.68)	<0.001***	15.8475 ± 2.2585	15.5857 ± 1.9095	0.388
TSH (μIU/mL)	1.62 (1.11,2.47)	2.00 (1.30,2.94)	<0.001***	5.89 (5.13,6.91)	5.40 (4.72,6.51)	0.026*
CysC (mg/L)	0.88 (0.77,1.03)	0.85 (0.73,1.00)	<0.001***	1.05 (0.83,1.41)	0.82 (0.71,1.02)	<0.001***
UA (umol/L)	321.50 (264.75,392.00)	293.00 (244.00,355.00)	<0.001***	350.6168 ± 99.9090	311.6159 ± 94.3460	0.007**
Cr (μmol/L)	67.95 (54.95,89.90)	52.50 (46.00,66.00)	<0.001***	72.25 (60.90,100.00)	53.65 (44.83,68.25)	<0.001***
CHO (mmol/L)	4.36 (3.69,5.06)	4.76 (4.02,5.38)	<0.001***	4.2194 ± 1.2276	4.6766 ± 1.1125	0.008**
Total cholesterol (μmol/dL)	214.81 (179.82,252.23)	226.61 (194.51,263.34)	<0.001***	229.7857 ± 60.6157	231.0751 ± 63.3592	0.889
TG (mmol/L)	1.41 (1.01,2.12)	1.52 (1.11,2.12)	0.01*	1.65 (1.06,2.15)	1.58 (1.10,2.19)	0.882
LDL-C (mmol/L)	2.80 (2.26,3.39)	2.98 (2.39,3.51)	<0.001***	3.1886 ± 1.0677	2.8547 ± 0.8714	0.018*
HDL-C (mmol/L)	1.06 (0.89,1.22)	1.18 (1.04,1.38)	<0.001***	0.9154 ± 0.2800	1.2249 ± 0.3192	<0.001***
Lp (a) (nmol/L)	100.65 (44.27,209.32)	119.30 (53.50,239.70)	<0.001***	117.40 (48.70,255.60)	133.25 (42.38,238.90)	0.708
Arteriosclerosis index	3.16 (2.47,3.77)	3.02 (2.35,3.59)	<0.001***	2.9065 ± 1.0530	2.9581 ± 0.9943	0.732
SGLT_2	173 (15.45%)	149 (12.05%)	0.016*	5 (6.94%)	10 (8.06%)	0.776
Insulin	274 (24.46%)	211 (17.06%)	0.001**	10 (13.89%)	22 (17.74%)	0.482
Ezetimibe	70 (6.25%)	54 (4.37%)	0.041*	7 (9.72%)	6 (4.84%)	0.185
Antihypertensive Agents	151 (13.48%)	243 (19.64%)	0.001**	14 (19.44%)	18 (14.52%)	0.368
HbA1c (%)	8.49 (7.10,9.60)	8.49 (6.90,8.70)	<0.001***	7.85 (6.78,8.49)	7.95 (6.67,8.49)	0.86
FRS	20.00 (17.00,22.00)	20.00 (17.00,22.00)	0.76	21.00 (16.75,24.00)	20.00 (16.00,22.00)	0.018*

BMI, body mass index; SBP/DBP, (systolic/diastolic blood pressure); FT3, free triiodothyronine; FT4, free thyroxine; TSH, Thyroid-Stimulating Hormone; CysC, Cystatin C; UA,Uric Acid; Cr, Creatinine; CHO, cholesterol; Total Cholesterol, Sum of triglycerides, LDL-C, and HDL-C; TG, Triglyceride; Lp(a), Lipoprotein(a); SGLT_2, Sodium-glucose cotransporter protein 2 inhibitor; HbA1c, Glycosylated Hemoglobin, Type A1C. *P<0.05, **P<0.01, ***P<0.001.

When comparing all male and female participants, male patients exhibited significantly elevated prevalence rates of smoking behavior and alcohol intake (both *P=* 0.001), DBP (*P=* 0.009), and BMI (*P=* 0.044); serum FT3, FT4, HbA1c, UA, Cr, CysC, atherosclerotic index, height, and weight (all *P<*0.001), use of insulin (*P=* 0.001), SGLT-2 inhibitors (*P=* 0.016), and ezetimibe (*P=* 0.041). In contrast, female patients demonstrated a significantly higher prevalence of SCH and hypertension (both *P=* 0.002), increased age and elevated SBP (both *P<*0.001), and more frequent use of antihypertensive medications (*P=* 0.001). Moreover, they exhibited significantly higher levels of serum TSH, CHO, LDL-C, HDL-C, and Lp(a) (all *P<*0.001), as well as TG (*P=* 0.01).

### 3.2 Ten-year cardiovascular disease risk by SCH status stratified by sex

**Figure 2:**
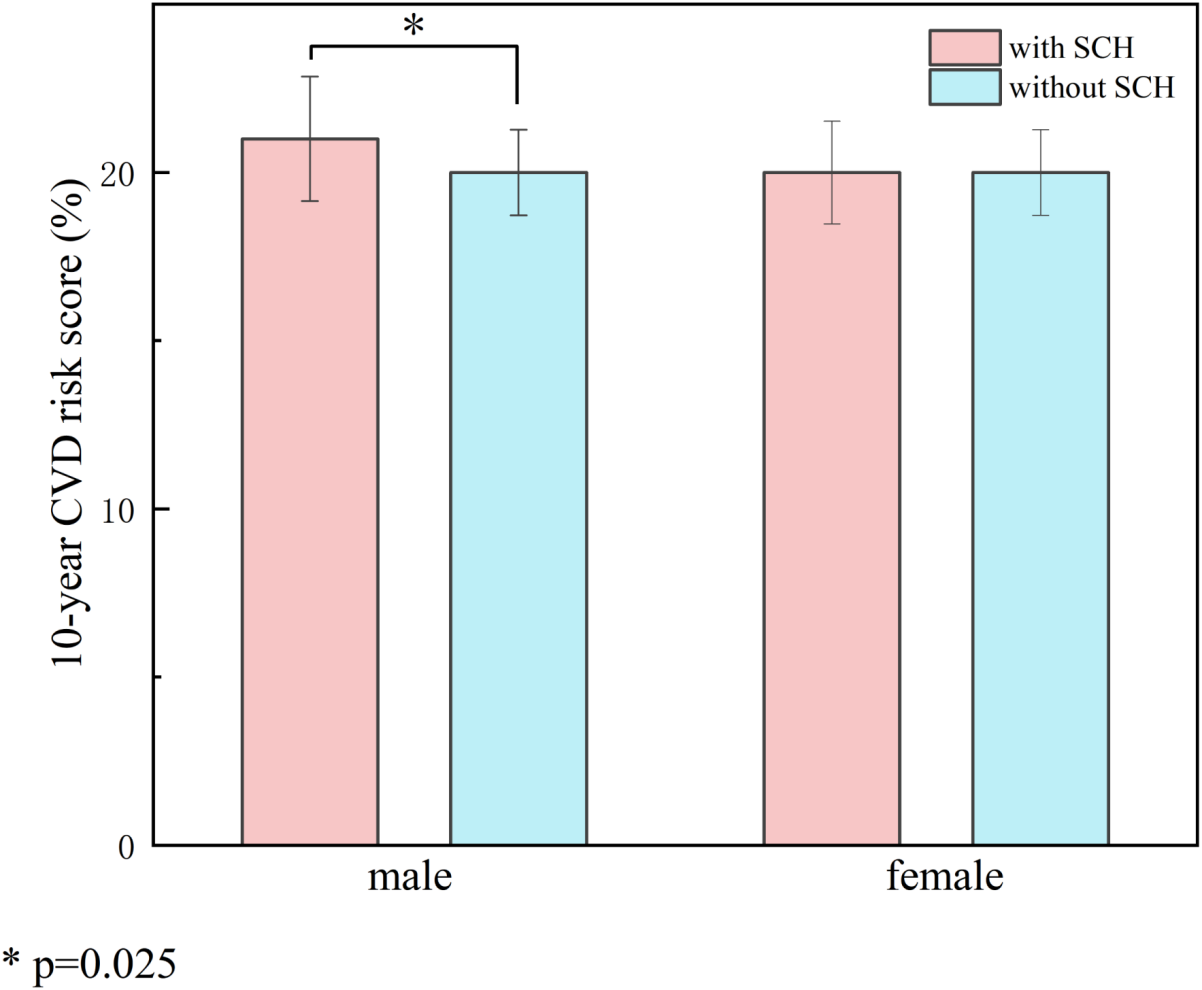
Impact of SCH on 10-year cardiovascular risk stratified by gender. Compares the 10-year CVD risk scores between patients with SCH and those with normal thyroid function, stratified by sex. The left panel represents the male group and the right panel the female group. Red bars indicate patients with SCH, and blue bars indicate euthyroid patients. The results showed that male patients with SCH had significantly higher FRS compared to euthyroid males (21.00 vs. 20.00, *P* = 0.025), whereas no significant difference was observed in the female group. The symbol * indicates a statistically significant difference with P = 0.025 (P < 0.05).

**Table 1** presents comparisons of the 10-year CVD risk, estimated using FRS, between patients with and without SCH, stratified by sex. Among male patients, the Framingham Risk Score was markedly elevated among individuals diagnosed with SCH relative to euthyroid controls (21.00 vs. 20.00, *P=* 0.025) (**Table 1**, **Figure 2**).

### 3.3 Correlation of TSH alongside CVD-related risk parameters with 10-year CVD risk by SCH status stratified by sex

**Figure 3:**
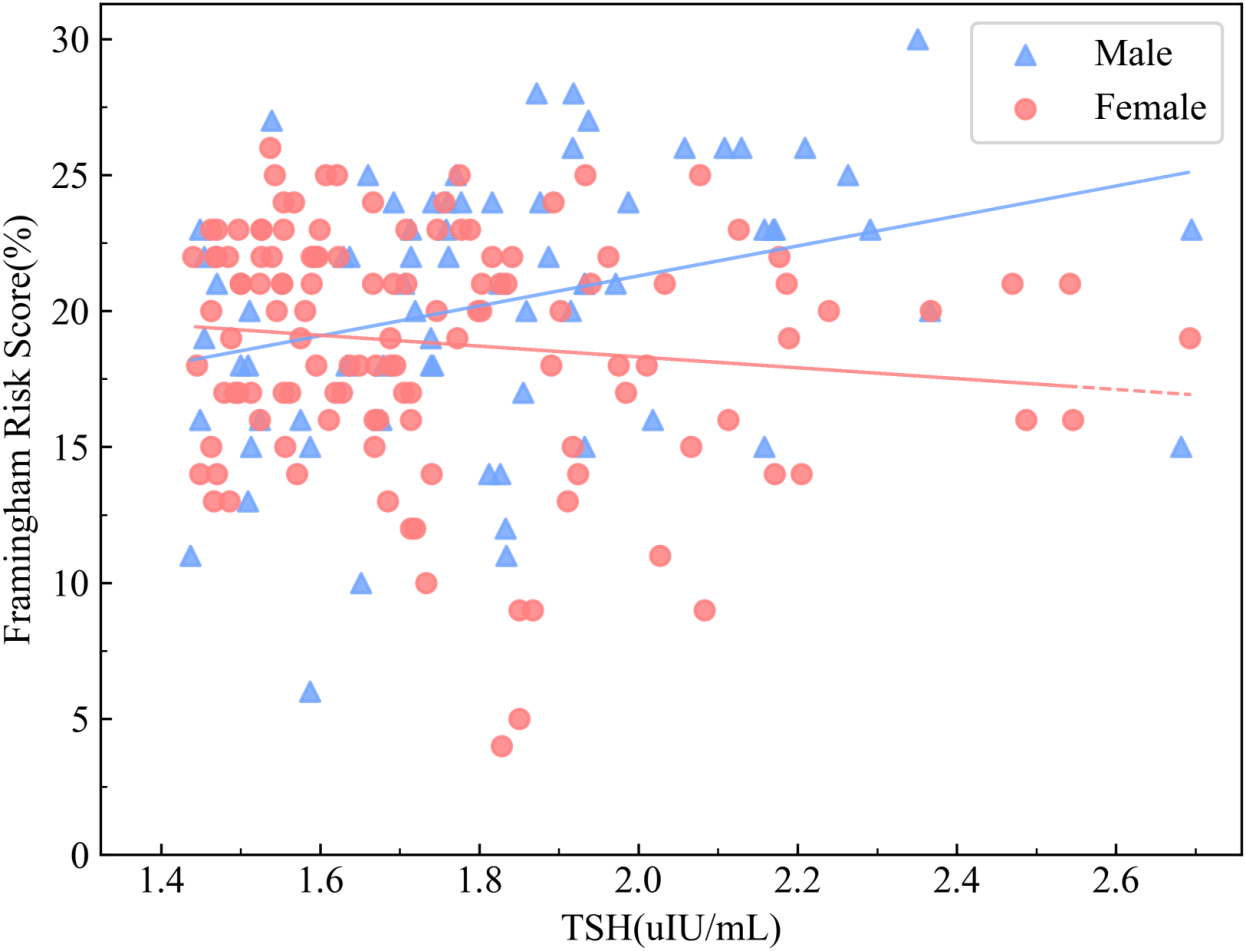
Correlation between serum TSH Levels and FRS stratified by sex in patients with DM and SCH. The association between TSH levels and FRS in patients with T2DM and SCH, stratified by sex, is shown. Blue triangles represent male patients with T2DM and SCH, while red circles represent female patients with DM and SCH. The results showed a significant positive correlation between TSH levels and FRS in male patients (*r* = 0.374, *P* = 0.001), whereas no significant association was observed in female patients (*r* = –0.142, *P* = 0.116).

Given the sex-specific variation in CVD risk, correlation analyses were conducted separately in the SCH groups of male and female patients (**Table 3**). In the male SCH group, serum TSH levels exhibited a significant positive correlation with FRS (*r=*0.374, *P=* 0.001). In contrast, the female SCH cohort demonstrated no statistically significant association(*r=*-0.142, *P=* 0.116) (**Figure 3**). FT4 demonstrated an inverse association with FRS among male individuals (*r=*-0.342, *P=* 0.003), but no meaningful relationship was identified in the female subgroup (*r=*0.022, *P=* 0.808). A significant direct association was observed between CysC levels and the estimated 10-year risk of cardiovascular disease among both male (*r=*0.461, *P<*0.001) and female patients (*r=*0.452, *P<*0.001).

**Table 3:** Correlation between thyroid-related hormones and additional clinical parameters with FRS in diabetic patients with SCH.

Variables	Male with SCH	P value	Female with SCH	P value
	Correlation coefficient		Correlation coefficient	
FT3 (pmol/L)	-0.171	0.151	-0.107	0.236
FT4 (pmol/L)	-0.342	0.003**	0.022	0.808
TSH (μIU/mL)	0.374	0.001**	-0.142	0.116
HbA1c (%)	-0.01	0.933	0.062	0.492
UA (μmol/L)	-0.03	0.806	0.22	0.014*
Cr (μmol/L)	0.098	0.415	0.372	<0.001***
CysC (mg/L)	0.461	<0.001***	0.452	<0.001***
LDL-C (mmol/L)	0.231	0.051	0.137	0.13
HDL-C (mmol/L)	0.012	0.922	-0.285	0.001**
Arteriosclerosis index	0.128	0.283	0.402	<0.001***
hsCRP (mg/L)	0.169	0.157	0.103	0.255

FT3, free triiodothyronine; FT4, free thyroxine; TSH, Thyroid-Stimulating Hormone; HbA1c, Glycosylated Hemoglobin, Type A1C; UA,Uric Acid; Cr, Creatinine; CysC, Cystatin C; LDL-C/HDL-C (low/high-density lipoprotein-cholesterol); hs-CRP, high-sensitivity C-reactive protein. *P<0.05, **P<0.01, ***P<0.001.

Based on multivariate regression modeling, elevated TSH (*β=*3.87, *P=* 0.048)and CysC(*β=*1.48, *P=* 0.03) levels, along with reduced FT4(*β=*-0.61, *P=* 0.011), emerged as significant independent predictors of 10-year CVD risk among males with SCH (**Table 4**). After adjusting for potential confounders, including alcohol consumption, use of statins, ezetimibe, SGLT2 inhibitors, insulin, and HbA1c, both TSH (*β* = 4.34, *P* = 0.027) and cystatin C (*β* = 1.66, *P* = 0.028) remained significantly positively associated with the 10-year Framingham cardiovascular risk score. Although free T4 did not reach statistical significance (*β* = –0.45, *P* = 0.071), its inverse association was consistent with the trend observed in the previous analysis. Insulin use was significantly negatively associated with FRS (*β* = –4.20, *P* = 0.020). Other variables, including alcohol consumption, statins, ezetimibe, and HbA1c, showed no significant associations (**Supplementary Table 3**).

**Table 4:** Multivariate linear regression analysis of factors associated with FRS in male patients with SCH.

Variables	Beta	SE	P value
TSH (μIU/mL)	3.870993	1.925889	0.048*
FT4 (pmol/L)	-0.613069	0.235561	0.011*
CysC (mg/L)	1.485689	0.66839	0.03*

TSH, Thyroid-Stimulating Hormone; FT4, free thyroxine; CysC, Cystatin C. *P<0.05.

Additionally, in male patients with SCH, a notable positive association was identified between serum TSH and CysC levels(*r=*0.305, *P=* 0.009) (**Table 5**).

**Table 5:** Correlation between TSH and cystatin C, FT4, FT4, smoking, uric acid, and creatinine in diabetic patients with SCH.

Variables	Male with SCH	P value	Female with SCH	P value
	Correlation coefficient		Correlation coefficient	
CysC(mg/L)	0.305	0.009**	0.043	0.634
FT4(pmol/L)	-0.286	0.015*	-0.075	0.406
Smoking	0.079	0.511	-0.108	0.231
UA(μmol/L)	0.015	0.904	-0.09	0.321
Cr(μmol/L)	0.208	0.08	0.017	0.854

CysC, Cystatin C; FT4, free thyroxine; UA, Uric Acid; Cr, Creatinine. *P<0.05, **P<0.01.

## 4 Discussion

A comprehensive study was conducted to investigate the association between SCH and 10-year cardiovascular risk among individuals with T2DM, with an emphasis on sex-specific differences. Our results indicated that SCH was markedly more common among female compared to their male counterparts(10.02% vs. 6.43%, respectively). Notably, male patients with comorbid SCH demonstrated markedly elevated 10-year CVD risk scores relative to their euthyroid counterparts. In this high-risk male subgroup, TSH, CysC, and FT4 levels emerged as significant biochemical correlates of increased CVD risk, underscoring the clinical imperative for early SCH screening and intervention in diabetic males to mitigate cardiovascular complications. **Graphical Abstract** visually summarizes the study’s design, analysis, and conclusions.

Our analysis further identified an overall SCH prevalence of 9.06% in diabetic patients—substantially higher than the 3.40% observed in non-diabetic populations (12), reinforcing the established comorbidity between diabetes and thyroid dysfunction. Mechanistically, diabetes-associated hyperleptinemia may dysregulate the hypothalamic-pituitary-thyroid (HPT) axis, potentiating TSH hypersecretion and SCH development (13, 14). Although both conditions independently elevate cardiovascular risk (1, 15), their synergistic impact remains controversial. While some studies report amplified CVD risk in T2DM-SCH comorbidity (4, 16), others found no significant association (5). Notably, our sex-stratified analysis revealed that SCH conferred excess 10-year CVD risk exclusively among males, whereas no comparable effect among females. This sexual dimorphism may be multifactorial: SCH-positive males exhibited higher smoking rates, UA, and Cr levels versus SCH-positive females (all *P<*0.05)—established CVD risk modifiers (17–19). Importantly, TSH, CysC, and FT4 levels specifically correlated with CVD risk in diabetic males with SCH, suggesting endocrine-renal interplay in driving this sex-specific vulnerability.

Evidence from previous investigations suggests that even minimal alterations in TSH levels can markedly impact the cardiovascular system in individuals with SCH (20), and elevated TSH concentrations have been linked to an increased risk of cardiovascular disease (21). Within the scope of this research, higher TSH values were linked to increased estimates of 10-year CVD risk among male patients presenting with concurrent DM and SCH. The observed association retained its significant association after controlling for potential confounding factors in the multivariate regression model. However, no statistically significant relationship was detected in female subjects. Moreover, male patients with DM and comorbid SCH exhibited significantly elevated TSH levels compared to their female counterparts, suggesting a potentially greater clinical relevance of TSH-related cardiovascular risk among men.

Moreover, a pooled analysis has indicated that higher serum concentrations of CysC correlate with a heightened risk of cardiovascular events (22). Among individuals diagnosed with T2DM, CysC is thought to contribute to cardiovascular risk through mechanisms involving insulin resistance and inflammatory pathways (23). Our analysis revealed a significant positive relationship between CysC and FRS among male patients with concurrent DM alongside SCH, and this association remained significant in multivariate regression models. Furthermore, comparative analysis revealed markedly greater CysC levels in male versus female patients with DM and comorbid SCH, supporting the notion that elevated CysC may confer greater cardiovascular risk in men.

It has been suggested that emerging evidence indicates that nephropathy-related pathways may underlie the elevated cardiovascular risk observed in individuals with T2DM and concurrent SCH (16). And elevated cystatin C levels, recognized as a sensitive indicator of kidney function, suggest renal impairment (24). In this study, a notable positive relationship was identified between TSH and CysC in male patients with DM complicated by SCH, supporting the hypothesis that elevated TSH may amplify cardiovascular risk in this population by exacerbating renal dysfunction (16, 25).

In addition, this study identified a significant inverse relationship between FT4 levels and FRS among male patients with SCH. This finding contradicts prior research, which had demonstrated an association between elevated FT4 levels and heightened cardiovascular risk (26). It should be emphasized that the study cited in (26) was conducted in a euthyroid population, whereas our analysis specifically targeted individuals with SCH, who may exhibit compensatory alterations in thyroid hormone metabolism. Several other studies lend support to the plausibility of our findings. For example, in middle-aged individuals exhibiting normal thyroid function, low FT4 levels have been correlated with elevated cardiovascular risk (27). A cross-sectional study also demonstrated a significant inverse relationship with coronary artery calcification among men, remaining significant after adjusting for conventional cardiovascular risk factors (28).

Furthermore, it has been proposed that the relationship between FT4 and CVD risk may follow a J-shaped curve, with the 20th to 40th percentiles of FT4 levels (Med. 13.5 pmol/L(IQR 11.2–13.9)) representing the optimal range for cardiovascular health (29). In this work, FT4 levels in male patients with SCH were found to be outside this proposed optimal range. Nevertheless, given the cross-sectional design, residual confounding factors, including thyroid autoantibody status and subclinical inflammation, cannot be excluded. Additionally, differences in assay standardization across studies may account for inconsistencies in FT4-related findings. Future prospective cohort studies are warranted to elucidate the mechanisms through which FT4 influences cardiovascular risk in patients with SCH. In addition, this study found that insulin use was significantly negatively associated with the FRS, which is consistent with previous findings (30), supporting the hypothesis that insulin therapy may be associated with a reduced cardiovascular risk.

Gender differences in cardiovascular risk among individuals with DM and SCH are well recognized but remain poorly understood. Earlier evidence suggests that among individuals with diabetes mellitus, the proportion of men classified as having very high cardiovascular risk surpasses that of women (55.6% vs. 50.7%) (31). In contrast, women with DM have been shown to be at increased risk of death due to cardiovascular autonomic neuropathy (32). Furthermore, a systematic review and meta-analysis of prospective studies with long-term follow-up (≥10 years) reported that men with SCH exhibited a markedly elevated probability of major adverse cardiovascular events (MACE) than women (33),whereas associations between SCH and cardiovascular risk scores have also been reported in women (34). However, gender-specific differences in cardiovascular risk among individuals with coexisting DM and SCH have not been well documented. Findings from this investigation demonstrated that the association between this comorbidity and elevated cardiovascular risk was more pronounced in male individuals than their female counterparts, suggesting a potential gender-specific effect. In summary, the observed gender disparities may be attributable to differences in smoking behavior, elevated levels of UA and Cr, and alterations in TSH, CysC, and FT4 levels.

Consistent with prior research (35, 36), a pronounced decline in HDL-C was detected in patients with concurrent DM and SCH relative to euthyroid controls. Moreover, males exhibited reduced HDL-C levels compared to females in both the overall population and the SCH subgroup, reinforcing the existence of gender disparities in lipid profiles (37, 38). However, in contrast to prior studies, no notable relationship was identified between HDL-C and FRS among men with comorbid DM and SCH. This discrepancy may be explained by the interplay of two mechanisms. First, elevated TSH may influence HDL-C subfractions by reducing the activity of CETP (cholesteryl ester transfer protein) and PLTP (phospholipid transfer protein), resulting in decreased HDL2-C and increased HDL3-C levels, which may alter cardiovascular risk profiles (39). Second, previous studies have reported significantly lower testosterone levels in male patients with SCH (40, 41), and low testosterone has been implicated in increased cardiovascular risk via endothelial dysfunction (42), potentially obscuring the independent relationship between HDL-C and FRS.

To the best of our understanding, this represents the first evidence of gender-specific variation in cardiovascular risk among patients with comorbid DM and SCH, highlighting a stronger association with decadal cardiovascular risk in men. The present results offer new insights for risk stratification and management in this patient population. Clinicians should be particularly vigilant in assessing cardiovascular risk among male patients with comorbid DM and SCH, as they may be at greater risk. However, the exact mechanism of the gender difference is unclear, and More investigations are required to explore the contribution of TSH, CysC, FT4, and other metabolic-related markers in this context. For male patients with T2DM and subclinical hypothyroidism (SCH), annual monitoring of TSH and cystatin C levels should be emphasized, in accordance with international guidelines (43) and the findings of the present study. Although this study identified a significant association between TSH levels and cardiovascular risk scores in male patients with T2DM and SCH, whether levothyroxine (LT4) therapy can improve cardiovascular outcomes remains controversial (44, 45). Given the cross-sectional observational design and the exclusion of patients with a history of LT4 use, this study cannot directly address the efficacy of LT4 treatment. Prospective interventional studies are warranted in male patients with T2DM and SCH to further evaluate the potential impact of LT4 therapy on cardiovascular outcomes.

Several limitations merit attention. Primarily, the cross-sectional design of the research precludes the establishment of causality. Second, our results’ applicability to broader populations may be restricted by the single-center setting, which may limit the generalizability of the findings due to regional, ethnic, and population differences. Variations in genetic background, lifestyle, and dietary habits across different regions and ethnicities may influence thyroid function as well as the mechanisms and risk of cardiometabolic diseases (44). Furthermore, studies investigating the relationship between hypothyroidism and hypertension in patients with diabetes have demonstrated substantial differences in cardiometabolic risk across populations. For example, individuals who are over 50 years old, female, of lower socioeconomic status, married, or with a family history of related conditions have been found to have a higher risk of both hypertension and diabetes (46). Third, although a broad range of clinical and biochemical indicators was included, thyroid autoantibodies and sex hormone levels (e.g., testosterone)—which are closely related to sex-specific differences in cardiovascular risk—were not measured. The absence of these variables may limit the interpretation of the underlying mechanisms. Future studies are recommended to incorporate these indicators to better elucidate the mechanisms contributing to sex-specific cardiovascular risk. Fourth, compared with imaging techniques such as angiography, the FRS predicts cardiovascular risk based on traditional risk factors. Furthermore, as a statistical model, the FRS provides probabilistic estimates, which may introduce classification bias compared to direct imaging methods. Such potential bias may arise from two aspects. First, the FRS was originally developed in White populations. However, a study based on a Taiwanese population found that female patients with SCH exhibited more pronounced region-specific fat deposition compared to European populations (47), suggesting possible limitations in the applicability of FRS across different ethnicities, regions, or specific subgroups. Moreover, previous research has demonstrated that among individuals classified as intermediate-risk by FRS, carotid plaque scores derived from ultrasound imaging can further identify those at genuinely high risk (48), indicating that FRS may underestimate cardiovascular risk in certain individuals. Given these limitations, the findings of the present study should be interpreted with caution. Future studies are encouraged to incorporate multiple assessment tools and modalities to provide a more comprehensive and accurate evaluation of cardiovascular risk.

## 5 Conclusion

This research suggests that male patients with comorbid T2DM and SCH exhibit significantly higher 10-year cardiovascular risk compared to their female counterparts. This sex-specific disparity appears to be driven by differences in both traditional cardiovascular risk factors (smoking status, serum UA, and Cr levels) and thyroid-renal biomarkers (TSH, CysC, and FT4). These results highlight the clinical urgency for sex-specific CVD risk stratification among patients with comorbid T2DM and SCH, with particular vigilance warranted for male populations. Specifically, integrating TSH and CysC monitoring into routine clinical practice may enhance early risk detection and personalized management. However, given the cross-sectional design, prospective cohort studies are essential to validate these observations and elucidate the causal mechanisms contributing to this sex-related disparity in cardiovascular vulnerability.

## Data Availability

The original contributions presented in the study are included in the article/**Supplementary Material**. Further inquiries can be directed to the corresponding authors.
