# Supplementary material for: Sex differences in 10-year cardiovascular risk of patients with type 2 diabetes mellitus and subclinical hypothyroidism: a cross-sectional study
Source: Front Endocrinol (Lausanne). 2025 Jul 31;16:1635444. doi: 10.3389/fendo.2025.1635444 (PMC12350142; doi:10.3389/fendo.2025.1635444)
Supplement: Supplementary file 1 [file Table1.docx]

Supplementary Material

# Supplementary Table 1. Comparison of Demographic and Clinical Characteristics With No Significant Differences Between Diabetic Patients With SCH and Those With Euthyroid Controls

| **Variables** | **Male** | |  | **Female** | |  |
| --- | --- | --- | --- | --- | --- | --- |
|  | **With SCH(N=72)** | **Without SCH(N=1048)** | ***P value*** | **With SCH(N=124)** | **Without SCH(N=1113)** | ***P value*** |
| Age(years) | 62.00(53.75,73.00) | 58.00(50.00,68.00) | 0.062 | 62.50(55.00,71.00) | 65.00(56.00,72.00) | 0.115 |
| Alcohol consumption | 7(9.72%) | 188(17.94%) | 0.075 | 0(0.00%) | 7(0.63%) | 0.376 |
| height(cm) | 170.00(167.00,173.25) | 170.00(168.00,175.00) | 0.268 | 160.00(155.00,163.00) | 160.00(156.00,163.00) | 0.491 |
| weight(kg) | 70.61(63.75,76.50) | 71.00(65.00,78.00) | 0.603 | 62.00(55.00,67.00) | 61.50(55.00,69.00) | 0.422 |
| BMI(kg/m^2^) | 24.28(22.48,26.32) | 24.46(22.49,26.37) | 0.953 | 24.06(22.06,25.98) | 24.12(22.22,26.37) | 0.582 |
| SBP(mmHg) | 135.50(124.00,150.00) | 132.00(125.00,143.00) | 0.266 | 137.93(125.75,150.00) | 138.00(126.00,150.00) | 0.961 |
| DBP(mmHg) | 82.74(75.00,90.00) | 80.00(76.00,90.00) | 0.833 | 80.00(76.75,90.00) | 80.00(75.00,86.00) | 0.641 |
| Hypertension | 38(52.78%) | 522(49.81%) | 0.626 | 61(49.19%) | 637(57.23%) | 0.087 |
| Fatty liver disease | 1(1.39%) | 48(4.58%) | 0.326 | 4(3.23%) | 48(4.31%) | 0.737 |
| FT3(pmol/L) | 4.58(4.15,4.94) | 4.53(4.03,4.96) | 0.464 | 4.19(3.81,4.65) | 4.20(3.74,4.66) | 0.55 |
| GLP-1 | 2(2.78%) | 22(2.10%) | 1 | 5(4.03%) | 28(2.52%) | 0.32 |
| Metformin | 32(44.44%) | 582(55.53%) | 0.067 | 64(51.61%) | 529(47.53%) | 0.388 |
| DPP-4 | - | - | - | 0(0.00%) | 7(0.63%) | 0.376 |
| Glinides | 5(6.94%) | 115(10.97%) | 0.285 | 12(9.68%) | 134(12.04%) | 0.439 |
| PPAR agonists | 3(4.17%) | 24(2.29%) | 0.544 | 3(2.42%) | 31(2.79%) | 1 |
| ACEI | 2(2.78%) | 30(2.86%) | 1 | 7(5.65%) | 43(3.86%) | 0.339 |
| ARB | 16(22.22%) | 244(23.28%) | 0.837 | 30(24.19%) | 294(26.42%) | 0.594 |
| β-blockers | 7(9.72%) | 121(11.55%) | 0.638 | 14(11.29%) | 140(12.58%) | 0.68 |
| Calcium channel blockers | 2(2.78%) | 27(2.58%) | 1 | 3(2.42%) | 37(3.32%) | 0.785 |
| Aspirin | 14(19.44%) | 151(14.41%) | 0.244 | 19(15.32%) | 166(14.91%) | 0.904 |
| Clopidogrel | 8(11.11%) | 142(13.55%) | 0.557 | 14(11.29%) | 153(13.75%) | 0.448 |
| Statins | 28(38.89%) | 297(28.34%) | 0.056 | 43(34.68%) | 338(30.37%) | 0.324 |
| Ezetimibe | 7(9.72%) | 63(6.01%) | 0.208 | 6(4.84%) | 48(4.31%) | 0.786 |
| Fibrates | - | - | - | 4(3.23%) | 30(2.70%) | 0.958 |
| UA(μmol/L) | 339.00(271.75,409.25) | 320.77(264.00,390.00) | 0.177 | 308.00(242.00,369.00) | 292.00(245.00,353.00) | 0.291 |
| CHO(mmol/L) | 4.12(3.46,4.86) | 4.37(3.73,5.08) | 0.059 | 4.83(3.98,5.33) | 4.75(4.03,5.40) | 0.573 |
| Total cholesterol(μmol/dL) | 224.09(190.26,257.35) | 214.23(179.33,251.84) | 0.217 | 225.83(186.97,258.61) | 226.61(194.90,263.34) | 0.386 |
| TG(mmol/L) | 1.65(1.06,2.15) | 1.40(1.00,2.11) | 0.258 | 1.58(1.10,2.19) | 1.51(1.12,2.12) | 0.773 |
| Lp(a)nmol/L | 117.40(48.70,255.60) | 99.85(43.95,206.25) | 0.517 | 133.25(42.38,238.90) | 118.20(54.30,239.71) | 0.883 |
| hs-CRP (mg/L) | 1.08(0.49,6.87) | 1.62(0.50,6.30) | 0.393 | 1.79(0.54,4.92) | 1.89(0.64,5.90) | 0.495 |
| Antihypertensive Agents | 2.89(2.01,3.62) | 3.16(2.48,3.78) | 0.056 | 2.94(2.25,3.60) | 3.03(2.36,3.59) | 0.434 |
| Use of antihypertensive medications | 14(19.44%) | 137(13.07%) | 0.126 | 18(14.52%) | 225(20.22%) | 0.13 |

BMI, body mass index; SBP, systolic blood pressure; DBP, diastolic blood pressure; FT3, free triiodothyronine; DPP-4: dipeptidyl peptidase-4; UA, uric acid; CHO, cholesterol; Total Cholesterol,Sum of triglycerides, LDL-C, and HDL-C; TG, triglycerides; Lp(a), lipoprotein(a); hs-CRP, high-sensitivity C-reactive protein; ACEI, angiotensin-converting enzyme inhibitor; ARB, angiotensin II receptor blocker; PPAR, peroxisome proliferator-activated receptor.

Supplementary Table 2. Comparison of Demographic and Clinical Characteristics With No Significant Differences Between Male and Female Diabetic Patients, and Between Male and Female Patients With SCH

| **Variables** | **Male（N=1120）** | **Female（N=1237）** | ***P value*** | **Male with SCH（N=72）** | **Female with SCH（N=124）** | ***P value*** |
| --- | --- | --- | --- | --- | --- | --- |
| Family history of coronary heart disease | 10(0.89%) | 6(0.49%) | 0.229 | - | - | - |
| Fatty liver disease | 49(4.38%) | 52(4.20%) | 0.838 | 1(1.39%) | 4(3.23%) | 0.752 |
| Metformin | 614(54.82%) | 644(52.06%) | 0.18 | 40(55.56%) | 64(51.61%) | 0.594 |
| DPP-4 | 2(0.18%) | 7(0.57%) | 0.235 | - | - | - |
| Glinides | 120(10.71%) | 146(11.80%) | 0.404 | 5(6.94%) | 12(9.68%) | 0.512 |
| AGIs | 372(33.21%) | 425(34.36%) | 0.558 | 25(34.72%) | 60(48.39%) | 0.063 |
| PPAR | 27(2.41%) | 34(2.75%) | 0.606 | 3(4.17%) | 3(2.42%) | 0.799 |
| ACEI | 32(2.86%) | 50(4.04%) | 0.117 | 2(2.78%) | 7(5.65%) | 0.568 |
| ARB | 260(23.21%) | 324(26.19%) | 0.094 | 16(22.22%) | 30(24.19%) | 0.754 |
| β-Blockers | 128(11.43%) | 154(12.45%) | 0.446 | 7(9.72%) | 14(11.29%) | 0.732 |
| Calcium channel blockers | 29(2.59%) | 40(3.23%) | 0.354 | 2(2.78%) | 3(2.42%) | 1 |
| Diuretics | 68(6.07%) | 90(7.28%) | 0.243 | 10(13.89%) | 8(6.45%) | 0.082 |
| Aspirin | 165(14.73%) | 185(14.96%) | 0.879 | 14(19.44%) | 19(15.32%) | 0.457 |
| Clopidogrel | 150(13.39%) | 167(13.50%) | 0.939 | 8(11.11%) | 14(11.29%) | 0.969 |
| Statins | 325(29.02%) | 381(30.80%) | 0.345 | 28(38.89%) | 43(34.68%) | 0.554 |
| Fibrates | 45(4.02%) | 34(2.75%) | 0.087 | 0(0.00%) | 4(3.23%) | 0.31 |
| hs-CRP (mg/L) | 1.54(0.50,6.35) | 1.89(0.62,5.84) | 0.076 | 1.08(0.49,6.87) | 1.79(0.54,4.92) | 0.541 |

DPP-4: dipeptidyl peptidase-4; AGIs: α-glucosidase inhibitors; PPAR: peroxisome proliferator-activated receptor; ACEI, angiotensin-converting enzyme inhibitor; ARB, angiotensin II receptor blocker; hs-CRP, high-sensitivity C-reactive protein;

Supplementary Table 3. Multivariate regression analysis of FRS after adjustment for confounding factors.

| **Variables** | **Beta** | **SE** | ***P value*** |
| --- | --- | --- | --- |
| Constant | 14.6293 | 6.3346 | 0.024* |
| TSH(μIU/mL) | 4.3384 | 1.9111 | 0.027* |
| FT4(pmol/L) | -0.4494 | 0.2444 | 0.071 |
| CysC(mg/L) | 1.6581 | 0.7384 | 0.028* |
| Alcohol consumption | 2.0778 | 1.7216 | 0.232 |
| Statins | 1.4385 | 1.2004 | 0.235 |
| Ezetimibe | -0.5761 | 1.8431 | 0.756 |
| SGLT_2 | 2.0044 | 2.0017 | 0.321 |
| Insulin | -4.1952 | 1.7622 | 0.02* |
| HbA1c(%) | 0.3137 | 0.3095 | 0.315 |

TSH: thyroid-stimulating hormone; FT4: free thyroxine; CysC: cystatin C; SGLT_2: sodium-glucose cotransporter-2 inhibitors; HbA1c,Glycosylated Hemoglobin, Type A1C; *P<0.05. Values with P < 0.05 were interpreted as statistically significant.
